# Factors associated with HIV testing among the general male population in Cambodia: A secondary data analysis of the Demographic Health Survey in 2005, 2010, and 2014

**DOI:** 10.1371/journal.pone.0219820

**Published:** 2019-07-18

**Authors:** Piseth Narin, Eiko Yamamoto, Yu Mon Saw, Ny Net, Souphalak Inthaphatha, Tetsuyoshi Kariya, Nobuyuki Hamajima

**Affiliations:** 1 Department of Healthcare Administration, Nagoya University Graduate School of Medicine, Nagoya, Japan; 2 National AIDS Authority, Phnom Penh, Cambodia; 3 Department of International Cooperation, Ministry of Health, Phnom Penh, Cambodia; National University of Singapore, SINGAPORE

## Abstract

In Cambodia, the human immunodeficiency virus (HIV) is predominantly transmitted between spouses and casual partners, with men having higher mortality and morbidity from HIV infection than women due to lesser access to healthcare services and antiretroviral therapy. This study aimed to identify the rate of HIV testing and barriers to HIV testing among the general male population in Cambodia. We analyzed secondary data of men who underwent HIV testing at Voluntary Confidential Counseling and Testing (VCCT) sites in 2006–2017 and of male participants in the Cambodia Demographic and Health Survey (CDHS) in 2005, 2010, and 2014. The number of men who underwent HIV testing at the VCCT sites increased during 2006–2010 and decreased during 2012–2015. CDHS data showed that the lifetime prevalence of HIV testing among men aged 15–49 years gradually increased from 14.7% in 2005 to 36.4% in 2014. Multivariate analysis revealed nine factors associated with a higher lifetime prevalence of HIV testing including: seven sociodemographic factors, namely CDHS year (2010 and 2014), age groups (20–35 and 36–49 years), urban residence, higher education, higher wealth index, having occupations other than agriculture, ever-married status (married and widowed/divorced); and two factors of HIV risk behavior, namely two or more lifetime sexual partners and condom use during the last sexual intercourse. To our knowledge, this is the first study that assessed factors associated with the lifetime prevalence of HIV testing among the general male population in Cambodia. The factors were mostly sociodemographic factors, and no factors were related to condom use, or the diagnosis or symptoms of sexually transmitted infections (STIs). These results suggest that reproductive health education at primary schools and strengthening of healthcare provider-initiated testing and counseling for patients with STIs are highly needed in Cambodia.

## Introduction

In Cambodia, the estimated prevalence of human immunodeficiency virus (HIV) infection in the general population aged 15–49 years reached a peak of 1.7% in 1998 and decreased to 0.6% in 2016 [1, 2]. Because HIV infection spread rapidly after the first reported case in 1991 [3], the Ministry of Health of Cambodia developed political strategies for preventing HIV infection and acquired immune deficiency syndrome (AIDS). The National Center for HIV/AIDS, Dermatology and STDs (NCHADS), which was established in 1998 following the merger of the National AIDS Programme and the National STD and Dermatology Clinic, developed HIV/AIDS strategic plans under its role as the responsible unit within the Ministry of Health.

The first strategic plan for 1993–1998 focused on the prevention of transmission by blood transfusion or condom use, including health education for the general and high-risk populations. The 100% Condom Use Programme was implemented in 1998 for preventing HIV infection and sexually transmitted infections (STIs) by providing condoms, education, and STI checkups to female entertainment workers (FEWs). In the second and third strategic plans for 1998–2000 and 2001–2005, respectively, care of people living with HIV and voluntary testing were added [4]. Key populations (KPs) at risk for HIV infection in Cambodia were reported to be men who have sex with men (MSM), people who use drugs, FEWs, transgender people, and prisoners [4–7]. However, not only KPs but also the general population have been included as the targets of some prevention strategies [4, 8]. According to the reports by NCHADS, the numbers of new HIV infections and deaths in people who have HIV infection have been higher in men than in women, although the total numbers of new infections and deaths have been decreasing [2].

Knowing HIV status is the first step towards eradicating AIDS. In 2014, the Joint United Nations Programme on HIV/AIDS (UNAIDS) set the target percentage of knowing HIV status among people living with HIV as 90% by 2020 and 95% by 2030 [9]. HIV voluntary confidential counseling and testing (VCCT) has been recommended for HIV infection prevention and an entrance for care and support for people living with HIV [10–12]. The first centers for VCCT were established in Phnom Penh and some provinces in 1995–1997 [13], before the first policy and guidelines for HIV testing and counseling were formally endorsed in 2002. NCHADS extended VCCT services to 255 VCCT sites in all 24 provinces by the end of 2011, which was supported by the government and health development partners [6, 14]. Three rapid diagnostic tests, namely Determine HIV-1/2, HIV 1/2 Stat-Pak, and Uni-Gold HIV-1/2, have been used as standard procedures of HIV testing at all VCCT sites.

To promote compliance with HIV testing and provide early HIV diagnosis, healthcare provider-initiated testing and counseling (HPITC) [15] was introduced in Cambodia in 2007 and has been completely assimilated since 2013 [6]. In this approach, healthcare providers can initiate VCCT for people who visit health facilities and would benefit from knowing their HIV infection status. The prevalence of HIV infection is high among sick people who visit health facilities, but many patients do not receive information regarding HIV infection or have an HIV test. HPITC provides systematic linkages between HIV testing and maternal and child health and tuberculosis (TB) programs [16] and testing is routinely offered to people who visit health facilities for STI checkup, drug treatment, and management of pediatric malnutrition [6].

The predominant mode of HIV transmission in Cambodia is evolving from sex work to transmission between spouses and casual partners [16, 17]. NCHADS reported that 74% of 4,254 people from 15 operational districts who were confirmed HIV positive in 2014–2016 comprised the general population [18, 19]. Therefore, it is important for the general population to undergo VCCT, not only for them to know their HIV infection status, but also for behavior change. Behavior change has been strongly associated with HIV infection prevalence and prevention of HIV infection by taking appropriate action [10, 20]. A meta-analysis of the effectiveness of VCCT in reducing behavioral risk in developing countries revealed that unprotected sex was reduced in people who had VCCT compared to before having VCCT or compared to people who had no VCCT [10]. Moreover, HIV mortality risk was reduced in people who tested positive, with a critical link to life-prolonging treatment [10, 21].

Most previous studies on HIV testing in Cambodia were conducted among KPs because they have been the high-risk groups for HIV infection. The prevalence of HIV testing was 79.9–81.7% in FEWs [22, 23], 83.6% in MSM [24], and 80.4% in transgender women [25]. Among the general population in Cambodia, the proportion of those receiving antiretroviral therapy (ART) was reported to be lower in men than women, and the mortality and morbidity from HIV infection have been higher in men than in women [1, 18]. Men are less likely to use healthcare services than women; therefore, a community-based HIV testing approach may be critical to men, including clients of entertainment venues, STI clients, partners of HIV positive women, and other high-risk men. However, there has been no study on factors associated with HIV testing among the general male population, although some studies have been conducted among the general population or the general female population [26–28]. This study aimed to identify the rate of HIV testing and barriers to HIV testing in the general male population. Findings from this study would support HIV programs in increasing the adoption of HIV testing to identify people living with HIV who are not aware of their HIV infection status, and link them to care and treatment that would in turn reduce further transmission through treatment-as-prevention strategy.

## Materials and methods

### Reporting system for VCCT in Cambodia

The number of men who underwent HIV testing at VCCT sites, the male population for each age group, and the number of HIV-positive men were extracted from VCCT data in each quarter of 2006–2017 [14]. VCCT data from the fourth quarter of 2005 were also available. The number of VCCT sites was 112 in the first quarter of 2006, which increased to 255 in 24 provinces in 2011 and then decreased to 70 in the fourth quarter of 2017. VCCT data were compiled at the operational district level and then at the provincial level. The Data Management Unit of NCHADS is responsible for consolidating and analyzing the nationwide data and disseminating them via the NCHADS website on a quarterly basis. VCCT data are also incorporated into the health information system of the Ministry of Health at all levels.

### The Cambodia Demographic and Health Survey data

The Cambodia Demographic and Health Survey (CDHS) is a national survey involving representative samples of women and men aged 15–49 years from all 24 provinces. The survey was approved by the National Ethics Committee for Health Research of Cambodia and the Institutional Review Boards of the collaborating agencies. The CDHS was implemented by the Directorate General for Health of the Ministry of Health, and the National Institute of Statistics of the Ministry of Planning with technical assistance by ORC International Inc. Samples were selected in two stages, and stratification was achieved by separating every reporting domain into urban and rural areas. Clusters (villages) were selected by applying probability proportional to village size in the first stage. In the second stage, households were selected in every urban and rural cluster based on the household listings, and the resulting sampling was corrected by applying sampling weights to the data, which ensured the validity of the sample for all strata such as urban and rural. Weights were calculated properly to guarantee the representativeness of the survey data and to prevent bias caused by nonresponse. Small areas and urban areas were oversampled, and this oversampling was corrected in the analysis using sampling weights to ensure the natural representation of the sample for all 38 strata (19 domains by urban or rural area). All men aged 15–49 years who lived in households of the subsample were included in the survey. The number of eligible men and the response rate were 7,229 and 93.1% in 2005, 8,665 and 95.1% in 2010, and 5,484 and 94.6% in 2014 [29–31].

CDHS data in 2005, 2010, and 2014 were used for this study because the same questionnaires related to HIV risk behaviors were used during these times. The number of male participants who completed the individual interview of the CDHS was 6,731 in 2005, 8,239 in 2010, and 5,190 in 2014 [29–31]. We used their sociodemographic information (age, residence, education, wealth index, occupation, and marital status), HIV risk behaviors (number of lifetime sexual partners, last sexual partner, second-to-last sexual partner, condom use during the last sexual intercourse with the partners, had any STI in the last 12 months, had genital sore/ulcer in the last 12 months, and had genital discharge in the last 12 months), and knowledge and coverage of prior HIV testing (knows a place to get an HIV test, ever been tested for HIV, and type of facility where the HIV test was taken). The question regarding condom use was “the last time you had sexual intercourse with the last sexual partner (or with the second-to-last sexual partner), was a condom used?” and it did not include any time frame or clarification of relationships.

### Statistical analysis

The sampling weights were included in all statistical analyses using CDHS data. A logistic regression model was used to estimate the odds ratios of having an HIV test and the 95% confidence intervals. In multivariate analysis, all available factors were used for adjustment, and a dummy variable was applied for different periods of the CDHS. A p-value of <0.05 was considered statistically significant. The analysis was performed using Statistical Package for the Social Sciences (SPSS) version 24.0 (IBM SPSS Inc.).

## Results

### Trend in number of men who had HIV testing at VCCT sites from 2006 to 2017

**Fig 1:**
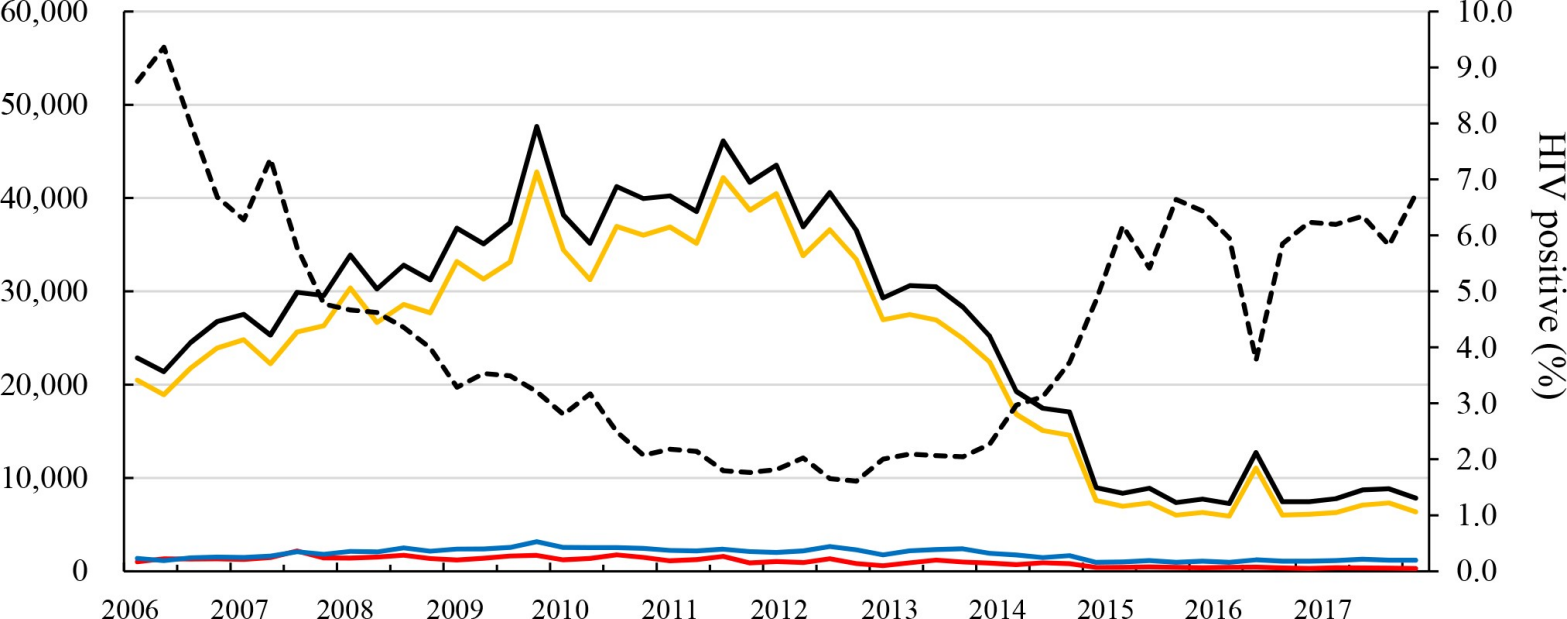
Number of Cambodian men who had an HIV test per quarter and HIV positive rate from 2006 to 2017. Solid black line = total number of men who had an HIV test; red line = aged <15 years; yellow line = aged 15–49 years; blue line = aged >49 years; dotted black line = HIV positive rate (%) of the total number of men who had an HIV test. HIV = human immunodeficiency virus.

The number of men who had an HIV test per quarter was approximately 23,000 in 2006, which increased to 47,661 in the first quarter of 2010. After the number plateaued at 35,000–46,000 between 2010 and 2012, it decreased gradually to 7,598 in the first quarter of 2015 and kept its pace until the fourth quarter of 2017 (Fig 1). Most men who had an HIV test at VCCT sites were 15–49 years old and the proportion of the age group ranged from 80.8% to 93.0%, with an average of 87.6%. The average proportion of the age group <15 years and >49 years was 4.1% (range 2.0–7.3%) and 8.3% (4.6–15.0%), respectively.

### Trend of HIV positive rate in men who had HIV testing at VCCT sites from 2006 to 2017

**Fig 2:**
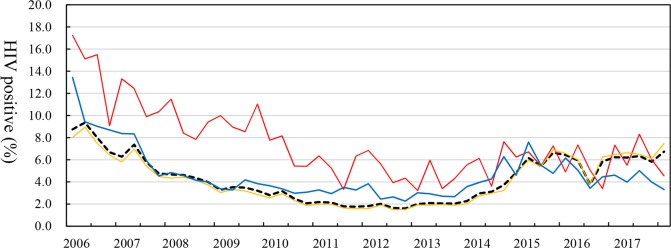
HIV positive rate of Cambodian men who had an HIV test at VCCT sites in each quarter between 2006 and 2017. Black dotted line = total number of men; red line = aged <15 years; yellow line = aged 15–49 years; blue line = aged >49 years. HIV = human immunodeficiency virus.

**Fig 3:**
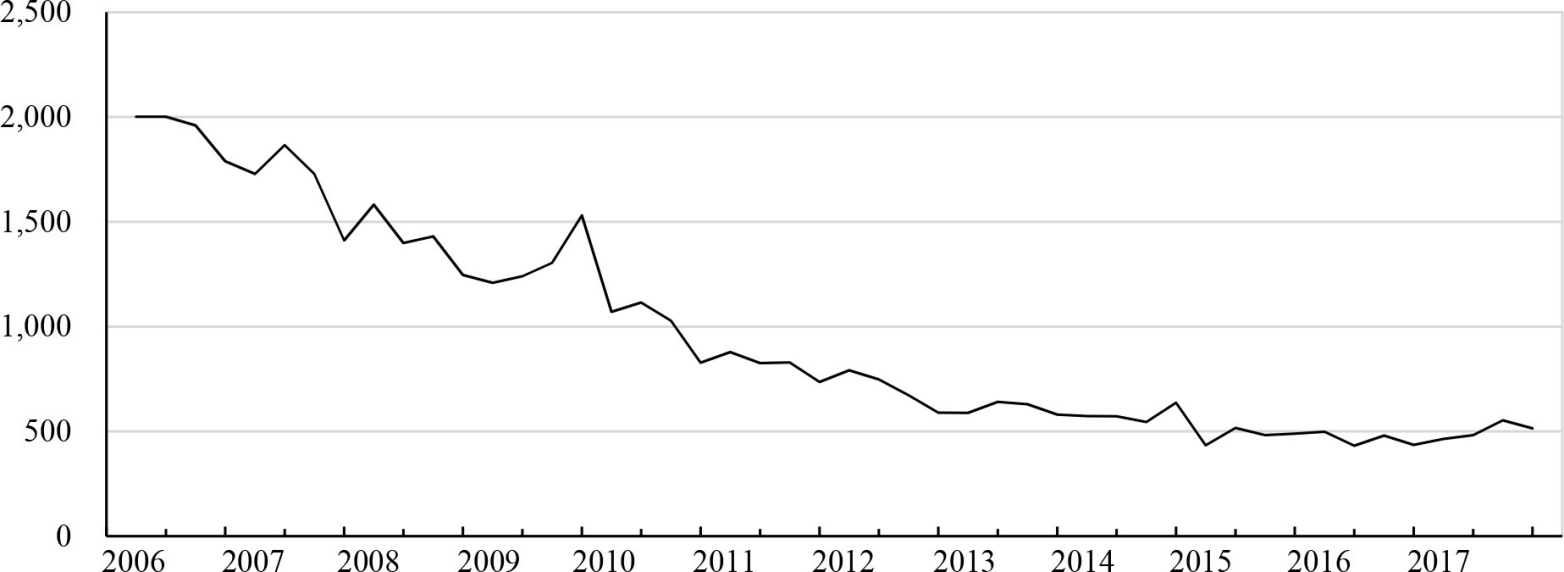
Number of men who tested positive for HIV. HIV = human immunodeficiency virus.

The HIV positive rate among men who had an HIV test was 8.7% and 9.4% in the first and second quarters of 2006, respectively, and decreased to 2.1% in the fourth quarter of 2010. The rate started increasing in 2014 and reached 6.2% in the second quarter of 2015. The HIV positive rate of the age group of <15 years was higher than those of the other groups. The difference in the positive rate between the age group of <15 years and the total men was 8.5% in the first quarter of 2006 and gradually decreased to 0–2% in 2015–2017 (Fig 2). Interestingly, the trend in the number of men who had an HIV test and the trend of the HIV positive rate were opposite (Fig 1). The number of HIV-positive men decreased from 2,001 in the first quarter of 2006 to 528 in the fourth quarter of 2017 (Fig 3).

### Sociodemographic characteristics and HIV risk history

To identify factors associated with HIV testing among men in Cambodia, we analyzed data on men aged 15–49 years who participated in the CDHS in 2005, 2010, and 2014. The total number of male participants was 20,160, which included 6,731 men in 2005, 8,239 in 2010, and 5,190 in 2014. For all years, the major age group was 20–35 years and the major area of residence was rural (Table 1). The proportions of men who had no education, who had no work, who were agriculture workers, and who had never married decreased from 2005 to 2014. Meanwhile, the proportions of men who had higher education and who were service workers or manual workers increased in the duration. Regarding HIV risk behaviors, in 2005–2014, the proportions of men who had three or more lifetime sexual partner and whose last sexual partner was FEW decreased. The proportion of men who had FEWs as the last-to-second sexual partner was decreased from 3.3% in 2005 to 0.5–0.8% in 2010–2014. The proportion of men who used condoms during their last sexual intercourse was 11.3% in 2005, which decreased to 8.0% in 2014. Only 0.3–0.9% of men had STIs or STI-related symptoms in each year. The proportion of men who knew a place for HIV testing increased from 46.7% in 2005 to 76.5% in 2014, and that of men who had ever had an HIV test also increased from 14.7% in 2005 to 36.4% in 2014. Most men had an HIV test at public health facilities, such as national hospitals, provincial hospitals, district hospitals, health centers, health posts, outreach centers, military hospitals, VCCT centers, and prevention of mother-to-child transmission sites, while 21.2–29.6% were tested at private facilities including private hospitals, clinics, laboratories, and mobile clinics.

**Table 1:** Characteristics of male participants of CDHS in 2005, 2010, and 2014.

Characteristics		2005 (N = 6731)	2010 (N = 8239)	2014 (N = 5190)	Total (N = 20160)
		n (%)	n (%)	n (%)	n (%)
Age (years old)					
	15–19	1662 (24.7)	1863 (22.6)	926 (17.8)	4451 (22.1)
	20–35	2863 (42.5)	3794 (46.0)	2557 (49.3)	9213 (45.7)
	36–49	2206 (32.8)	2582 (31.3)	1708 (32.9)	6496 (32.2)
Residence					
	Urban	1133 (16.8)	1697 (20.6)	869 (16.7)	3699 (18.3)
	Rural	5598 (83.2)	6542 (79.4)	4321 (83.3)	16461 (81.7)
Education					
	No education	606 (9.0)	641 (7.8)	324 (6.2)	1571 (7.8)
	Primary	3261 (48.4)	3394 (41.2)	2167 (41.8)	8821 (43.8)
	Secondary	2639 (39.2)	3681 (44.7)	2304 (44.4)	8624 (42.8)
	Higher	225 (3.3)	524 (6.4)	395 (7.6)	1144 (5.7)
Wealth index					
	Poorest	1078 (16.0)	1454 (17.6)	901 (17.4)	3433 (17.0)
	Poorer	1218 (18.1)	1544 (18.7)	954 (18.4)	3717 (18.4)
	Middle	1351 (20.1)	1637 (19.9)	1040 (20.0)	4029 (20.0)
	Richer	1468 (21.8)	1696 (20.6)	1124 (21.7)	4288 (21.3)
	Richest	1616 (24.0)	1908 (23.2)	1171 (22.6)	4694 (23.3)
Occupation					
	No work	1153 (17.1)	1079 (13.1)	409 (7.9)	2640 (13.1)
	Full-time worker	832 (12.4)	1024 (12.4)	719 (13.9)	2575 (12.8)
	Agriculture worker	3532 (52.5)	4233 (51.4)	2465 (47.5)	10229 (50.7)
	Service	212 (3.1)	310 (3.8)	307 (5.9)	829 (4.1)
	Manual	1003 (14.9)	1593 (19.3)	1290 (24.9)	3886 (19.3)
Marital status					
	Never married	2668 (39.6)	3255 (39.5)	1685 (32.5)	7608 (37.7)
	Married	3973 (59.0)	4852 (58.9)	3405 (65.6)	12231 (60.7)
	Widowed/divorced	89 (1.3)	132 (1.6)	100 (1.9)	321 (1.6)
Number of lifetime sexual partners					
	≤1	4727 (70.2)	6210 (75.4)	3896 (75.1)	14833 (73.6)
	2	570 (8.5)	656 (8.0)	504 (9.7)	1729 (8.6)
	≥3	1425 (21.2)	1368 (16.6)	789 (15.2)	3582 (17.8)
Last sexual partner					
	Spouse	3931 (58.4)	4778 (58.0)	3356 (64.7)	12065 (59.8)
	FEW	194 (2.9)	95 (1.2)	32 (0.6)	320 (1.6)
	Others	2606 (38.7)	3366 (40.9)	1802 (34.7)	7775 (38.6)
Second-to-last sexual partner					
	Spouse	29 (0.4)	25 (0.3)	13 (0.2)	66 (0.3)
	FEW	221 (3.3)	41 (0.5)	43 (0.8)	305 (1.5)
	Others	6481 (96.3)	8174 (99.2)	5134 (98.9)	19789 (98.2)
Condom use during the last sexual intercourse					
	No	3795 (88.7)	4597 (90.2)	3284 (92.0)	11675 (90.2)
	Yes	482 (11.3)	501 (9.8)	287 (8.0)	1271 (9.8)
	Don’t know	2454	3141	1619	7211
Condom use during the last sexual intercourse with the second-to-last sexual partner					
	No	83 (20.6)	43 (33.6)	39 (28.1)	165 (24.7)
	Yes	319 (79.4)	85 (66.4)	100 (71.9)	504 (75.3)
	Don’t know	6329	8111	5050	19486
Had any STI in the last 12 months					
	No	6704 (99.6)	8216 (99.7)	5168 (99.6)	20088 (99.6)
	Yes	27 (0.4)	23 (0.3)	22 (0.4)	72 (0.4)
Had genital sore/ulcer in the last 12 months					
	No	6697 (99.5)	8188 (99.4)	5153 (99.3)	20039 (99.4)
	Yes	34 (0.5)	51 (0.6)	37 (0.7)	121 (0.6)
Had any genital discharge in the last 12 months					
	No	6673 (99.1)	8190 (99.4)	5160 (99.4)	20023 (99.3)
	Yes	58 (0.9)	49 (0.6)	30 (0.6)	137 (0.7)
Know a place to get HIV test					
	No	3589 (53.3)	2532 (30.7)	1218 (23.5)	7339 (36.4)
	Yes	3142 (46.7)	5707 (69.3)	3972 (76.5)	12821 (63.6)
Ever been tested for HIV					
	No	5741 (85.3)	6192 (75.2)	3300 (63.6)	15233 (75.6)
	Yes	990 (14.7)	2047 (24.8)	1890 (36.4)	4927 (24.4)
Type of facility where HIV test taken^a^					
	Public^b^	622 (63.0)	1530 (74.9)	1331 (70.4)	3480 (70.7)
	Private^c^	277 (28.2)	435 (21.2)	559 (29.6)	1268 (25.7)
	Others^d^	87 (8.8)	81 (4.0)	1 (0.1)	169 (3.4)
	Had no test	5741	6192	3300	15233

Abbreviations: FEW, female entertainment worker; HIV, human immunodeficiency virus; STI, sexually transmitted infection.

^a^Percentages indicate the proportions among men who have ever taken an HIV test.

^b^Public facilities include national hospital, provincial hospital, district hospital, health center, health post, outreach, military hospital, VCCT center, prevention of mother-to-child transmission site, and other public sectors.

^c^Private facilities include private hospital, private clinic, private laboratory, private mobile clinic, and other private medical sectors.

^d^Others include home and correctional facilities.

### Factors associated with HIV testing among Cambodian men in 2005, 2010, and 2014

Multivariable logistic regression analyses were performed to identify factors associated with the lifetime prevalence of HIV testing among Cambodian men who participated in the CDHS in 2005, 2010, and 2014. Age, education, wealth index, occupation, marital status, number of lifetime sexual partners, and condom use during the last sexual intercourse were significantly associated with having an HIV test in all three years (Table 2). The results showed that men who were the age groups of 20–35 and 36–49 years old, who had high and very high wealth indices, who were ever married, who had a greater number of lifetime sexual partners, and who used condoms during the last sexual intercourse had a higher prevalence of having an HIV test. Men who resided in rural areas showed a lower prevalence of having an HIV test, but only the results for 2005 and 2010 showed a significant difference. The occupation results showed that full-time workers and those in the service or manual industries had a higher prevalence compared to those men who were not working in all years, whereas there was no difference between agriculture workers and men who had no working. In variables of HIV risk behavior, the associations of the last sexual partner, the second-to-last sexual partner, having an STI, and having genital discharge with the lifetime prevalence of having an HIV test were not consistent in the three CDHS years.

**Table 2:** Multivariable logistic regression analysis on HIV testing among male participants of CDHS 2005, 2010, and 2014.

Variables		CDHS 2005	CDHS 2010	CDHS 2014
		AOR^b^ (95% CI)	AOR^b^ (95% CI)	AOR^b^ (95% CI)
Age (years old)				
	15–19	1 (Reference)	1 (Reference)	1 (Reference)
	20–35	3.85 (2.61–5.68)***	5.31 (3.91–7.22)***	2.99 (2.18–4.08)***
	36–49	2.36 (1.54–3.62)***	2.17 (1.55–3.03)***	1.22 (0.87–1.73)
Residence				
	Urban	1 (Reference)	1 (Reference)	1 (Reference)
	Rural	0.77 (0.63–0.94)*	0.69 (0.58–0.83)***	0.82 (0.65–1.02)
Education				
	No education	1 (Reference)	1 (Reference)	1 (Reference)
	Primary	1.58 (1.10–2.28)*	1.49 (1.13–1.96)**	1.94 (1.41–2.67)***
	Secondary	2.03 (1.40–2.95)***	3.21 (2.43–4.25)***	2.90 (2.09–4.02)***
	Higher	3.43 (2.12–5.57)***	5.09 (3.51–7.37)***	4.40 (2.87–6.75)***
Wealth Index				
	Poorest	1 (Reference)	1 (Reference)	1 (Reference)
	Poorer	1.07 (0.75–1.53)	1.47 (1.17–1.84)**	0.99 (0.78–1.24)
	Middle	1.41 (1.01–1.97)*	1.66 (1.33–2.07)***	1.11 (0.88–1.39)
	Richer	2.12 (1.54–2.91)***	1.96 (1.56–2.45)***	1.72 (1.37–2.16)***
	Richest	2.93 (2.09–4.11)***	2.38 (1.83–3.09)***	2.32 (1.77–3.04)***
Occupation				
	No working	1 (Reference)	1 (Reference)	1 (Reference)
	Full-time worker	2.13 (1.50–3.01)***	2.72 (2.01–3.69)***	1.22 (0.85–1.77)
	Agriculture worker	0.96 (0.68–1.37)	1.36 (1.00–1.86)	0.97 (0.68–1.40)
	Service	3.45 (2.24–5.30)***	3.54 (2.43–5.14)***	1.90 (1.25–2.90)**
	Manual	1.45 (1.02–2.06)*	2.38 (1.75–3.24)***	1.26 (0.88–1.81)
Marital Status				
	Never married	1 (Reference)	1 (Reference)	1 (Reference)
	Married	2.95 (1.56–5.59)**	3.07 (1.96–4.79)***	4.72 (2.74–8.14)***
	Windowed/divorced	4.10 (2.37–7.10)***	5.44 (3.53–8.37)***	5.36 (3.28–8.77)***
Number of lifetime sexual partners				
	≤1	1 (Reference)	1 (Reference)	1 (Reference)
	2	1.75 (1.35–2.27)***	1.88 (1.55–2.29)***	1.67 (1.35–2.05)***
	≥3	2.74 (2.28–3.30)***	2.33 (2.01–2.70)***	2.54 (2.10–3.08)***
Last sexual partner				
	Spouse	1 (Reference)	1 (Reference)	1 (Reference)
	FEW	1.19 (0.57–2.45)	0.57 (0.29–1.12)	0.40 (0.14–1.13)
	Others	1.60 (0.87–2.95)	0.86 (0.56–1.31)	0.82 (0.49–1.37)
Second-to-last sexual partner				
	Spouse	1 (Reference)	1 (Reference)	1 (Reference)
	FEW	0.44 (0.15–1.29)	0.30 (0.08–1.15)	1.27 (0.27–5.99)
	Others	0.65 (0.25–1.65)	0.40 (0.15–1.10)	1.77 (0.47–6.65)
Condom use during the last sexual intercourse				
	No^a^	1 (Reference)	1 (Reference)	1 (Reference)
	Yes	1.44 (1.08–1.93)*	1.32 (1.04–1.67)*	1.47 (1.09–1.98)*
Condom use during the last sexual intercourse with the second-to-last sexual partner				
	No^a^	1 (Reference)	1 (Reference)	1 (Reference)
	Yes	1.24 (0.79–1.96)	1.65 (0.83–3.27)	1.35 (0.74–2.47)
Had any STI in the last 12 months				
	No	1 (Reference)	1 (Reference)	1 (Reference)
	Yes	4.51 (1.73–11.75)**	0.55 (0.18–1.67)	1.16 (0.37–3.62)
Had genital sore/ulcer in the last 12 months				
	No	1 (Reference)	1 (Reference)	1 (Reference)
	Yes	1.05 (0.42–2.62)	1.09 (0.54–2.19)	1.08 (0.47–2.44)
Had any genital discharge in the last 12 months				
	No	1 (Reference)	1 (Reference)	1 (Reference)
	Yes	0.82 (0.36–1.87)	2.09 (1.07–4.08)*	1.41 (0.57–3.45)

Abbreviations: AOR, adjusted odds ratio; CI, confidence interval; FEW, female entertainment worker; CDHS, Cambodia Demographic Health Survey; HIV, human immunodeficiency virus; STI, sexually transmitted infection.

* P<0.05

** P<0.01

*** P<0.001.

^a^Men who answered “no” and “don’t know.”

^b^Adjusted for age, residence, education, wealth index, occupation, marital status, number of lifetime sexual partners, last sexual partner, second-to-last sexual partner, condom use during the last sexual intercourse, condom use during the last sexual intercourse with the second-to-last sexual partner, had any STI in the 12 months, had genital sore/ulcer in the last 12 months, and had any genital discharge in the last 12 months.

### Factors associated with HIV testing among Cambodian men in 2005–2014

To reveal the factors associated with having an HIV test in their lifetime among men in 2005–2014, we performed logistic regression analysis using data on 20,160 men who participated in the three CDHS years. Univariate analysis showed that all variables were associated with the lifetime prevalence of HIV testing. However, multivariate analysis showed that the CDHS years of 2010 and 2014, the age groups of 20–35 and 36–49 years, urban residence, higher education, higher wealth index, having occupation other than agriculture, being married and widowed/divorced, having two or more lifetime sexual partners, condom use during the last sexual intercourse were significantly associated with a higher prevalence of having an HIV test (Table 3). Men whose last or second-to-last sexual partners were spouses, and who had diagnosis of STIs or STI-related symptoms in the last 12 months were more likely to have HIV testing but the difference was not significant in the multivariable analysis.

**Table 3:** Logistic regression analysis on HIV testing among all males respondents of CDHS 2005, 2010, and 2014.

Variables		Had HIV test		
		n (%)	OR (95% CI)	AOR^b^ (95% CI)
CDHS Year				
	2005	990 (14.7)	1 (Reference)	1 (Reference)
	2010	2047 (24.8)	1.92 (1.76–2.09)***	2.24 (2.03–2.48)***
	2014	1890 (36.4)	3.32 (3.04–3.63)***	3.86 (3.48–4.29)***
Age (years old)				
	15–19	169 (3.8)	1 (Reference)	1 (Reference)
	20–35	3084 (33.5)	12.76 (10.87–14.97)***	4.04 (3.34–4.89)***
	36–49	1674 (25.8)	8.80 (7.47–10.37)***	1.86 (1.51–2.29)***
Residence				
	Urban	1544 (41.7)	Reference	Reference
	Rural	3382 (20.5)	0.36 (0.34–0.39)***	0.75 (0.67–0.84)***
Education				
	No education	165 (10.5)	1 (Reference)	1 (Reference)
	Primary	1618 (18.3)	1.91 (1.61–2.26)***	1.68 (1.41–2.02)***
	Secondary	2516 (29.2)	3.50 (2.96–4.14)***	2.78 (2.31–3.34)***
	Higher	629 (54.9)	10.33 (8.47–12.60)***	4.21 (3.31–5.35)***
Wealth Index				
	Poorest	437 (12.7)	1 (Reference)	1 (Reference)
	Poorer	605 (16.3)	1.33 (1.17–1.52)***	1.18 (1.02–1.36)*
	Middle	751 (18.6)	1.57 (1.38–1.78)***	1.37 (1.19–1.57)***
	Richer	1148 (26.8)	2.50 (2.22–2.83)***	1.87 (1.63–2.15)***
	Richest	1986 (42.3)	5.02 (4.47–5.64)***	2.48 (2.12–2.91)***
Occupation				
	Not working	224 (8.5)	1 (Reference)	1 (Reference)
	Full-time worker	1239 (48.1)	10.01 (8.56–11.72)***	1.96 (1.61–2.37)***
	Agriculture worker	1651 (16.1)	2.08 (1.79–2.40)***	1.09 (0.90–1.33)
	Service	453 (54.6)	12.96 (10.68–15.72)***	2.76 (2.18–3.48)***
	Manual	1360 (35.0)	5.81 (4.99–6.76)***	1.66 (1.37–2.02)***
Marital Status				
	Never married	893 (11.7)	1 (Reference)	1 (Reference)
	Married	3896 (31.9)	3.51 (3.25–3.80)***	3.38 (2.50–4.57)***
	Windowed/divorced	137 (42.7)	5.61 (4.45–7.07)***	5.13 (3.88–6.78)***
Number of lifetime sexual partners				
	≤1	2564 (17.3)	1 (Reference)	1 (Reference)
	2	627 (36.3)	2.72 (2.45–3.03)***	1.73 (1.53–1.96)***
	≥3	1732 (48.4)	4.48 (4.15–4.85)***	2.46 (2.23–2.71)***
Last sexual partner				
	Spouse	3829 (31.7)	1 (Reference)	1 (Reference)
	FEW	110 (34.3)	1.12 (0.89–1.42)	0.81 (0.54–1.22)
	Others	988 (12.7)	0.31 (0.29–0.34)***	0.93 (0.70–1.24)
Second-to-last sexual partner				
	Spouse	33 (50.0)	1 (Reference)	1 (Reference)
	FEW	119 (39.0)	0.64 (0.37–1.09)	0.51 (0.26–1.03)
	Others	4775 (24.1)	0.32 (0.20–0.52)***	0.70 (0.38–1.26)
Condom use during the last sexual intercourse				
	No^a^	4368 (23.1)	1 (Reference)	1 (Reference)
	Yes	558 (43.9)	2.61 (2.32–2.93)***	1.43 (1.23–1.67)***
Condom use during the last sexual intercourse with the second-to-last sexual partner				
	No^a^	4687 (23.8)	1 (Reference)	1 (Reference)
	Yes	240 (47.6)	2.90 (2.43–3.47)***	1.37 (1.00–1.88)
Had any STI in the last 12 months				
	No	4889 (24.3)	1 (Reference)	1 (Reference)
	Yes	38 (52.8)	3.46 (2.18–5.50)***	1.51 (0.84–2.71)
Had genital sore/ulcer in the last 12 months				
	No	4880 (24.4)	1 (Reference)	1 (Reference)
	Yes	46 (38.3)	1.94 (1.34–2.80)***	1.07 (0.68–1.69)
Had any genital discharge in the last 12 months				
	No	4876 (24.4)	1 (Reference)	1 (Reference)
	Yes	50 (36.5)	1.79 (1.26–2.53)**	1.40 (0.91–2.16)

Abbreviations: AOR, adjusted odds ratio; CI, confidence interval; FEW, female entertainment worker; CDHS, Cambodia Demographic Health Survey; HIV, human immunodeficiency virus; OR, odds ratio; STI, sexually transmitted infection.

* P<0.05

** P<0.01

*** P<0.001.

^a^Men who answered “no” and “don’t know.”

^b^Adjusted for DHS year, age, residence, education, wealth index, occupation, marital status, number of lifetime sexual partners, last sexual partner, second-to-last sexual partner, condom use during the last sexual intercourse, condom use during the last sexual intercourse with the second-to-last sexual partner, had any STI in the 12 months, had genital sore/ulcer in the last 12 months, and had any genital discharge in the last 12 months.

## Discussion

This study showed the trend of HIV testing at VCCT sites and the lifetime prevalence of HIV testing among the general male population in Cambodia. The total number of men who had HIV testing in each quarter increased from 2006 to 2010 and decreased from 2012 to 2015. The CDHS data showed that the lifetime prevalence of HIV testing among men aged 15–49 years significantly increased from 2005 to 2014, ranging from 14.7% to 36.4%. The introduction of community-based testing programs might be one of the reasons for this increase in the lifetime prevalence [32–34]. Although most of these programs target KPs, the general population may account for some proportions of the clients. Several previous studies in KPs have consistently shown reduced HIV testing at VCCT sites and increased rates in peer-initiated testing in the community [2, 23, 24, 32]. People can also have an HIV test in private sectors and not in VCCT sites; in the survey, 25.7% of participants of CDHS 2005–2014 answered they were tested at private facilities.

To our knowledge, this is the first report about factors associated with HIV testing among the general male population in Cambodia. Multivariate analysis of three sets of CDHS data revealed nine factors associated with a higher lifetime prevalence of HIV testing. Of the nine factors, seven were sociodemographic factors, including the age groups of 20–35 and 36–49 years, urban residence, higher education, higher wealth index, having occupation other than agriculture, and an ever-married status. These results are consistent with those of previous studies that were conducted among the general population and KPs in developing countries [22, 35–37]. One study reported that more women with partners having a higher educational level underwent HIV testing in Cambodia, which suggests that the educational level of men is important because it was associated with a higher prevalence of HIV testing among men as well as women [26].

The two remaining factors included having two or more lifetime sexual partners and condom use during the last sexual intercourse. A higher number of sexual partners was reported to be associated with having an HIV test and with HIV infection prevalence in the general population and MSM [38, 39]. The results of the positive association between condom use and having an HIV test were consistent with those of studies among the general population in developing countries and among TB patients and MSM in Cambodia [10, 24, 25, 40], although a negative association between condom use with a noncommercial partner and having an HIV test was found in Cambodian FEWs [22]. In terms of sexual behavior, the results of this study suggest that Cambodian men who used condoms in the last sexual intercourse might be highly cautious about HIV infection compared to men who were nonusers of condoms. Men who used condoms during the last sexual intercourse with the second-to-last sexual partner were also more likely to have more HIV testing than the others, although the difference was not significant.

Interestingly, factors related to STIs were not significantly associated with lifetime HIV testing in the multivariable analysis of CDHS 2005–2014. Men who were diagnosed with any STI in CDHS 2005 and men who had genital discharge in CDHS 2010 had a significantly higher prevalence of HIV testing than the others. However, all three variables related to STIs were not associated with the lifetime prevalence of HIV testing in 2014. HIV infection prevalence is higher among STI patients, with a risk of 2.0–23.5 [41–43]. Genital ulcers increase HIV transmission by bleeding frequently during sexual intercourse, and nonulcerative STIs, such as gonorrhea and chlamydia, also increase genital shedding of HIV [41, 43]. FEWs and MSM who were diagnosed with STIs in the last 3–6 months were more likely to undergo HIV testing in the last 6 months [22, 24]. This study revealed that the general male population in Cambodia did not worry about having an HIV infection so much when they had genital sore or ulcer. Thus, the HPITC approach should be emphasized among STI patients, and since 93.7% of men had at least a primary level of education in 2014, education of reproductive health should be provided in primary schools to decrease the rate of STI transmission as well as HIV. School-based HIV prevention programs should be designed to be included as a part of school curriculum, targeting younger children and using highly qualified health-related textbooks as well as participatory activities, such as role plays, skits, and songs [44, 45].

In terms of the HIV positive rate at VCCT sites, the rate among the total men was high in 2006, but it might not represent the rate in the general population because the clients of HIV testing at VCCT sites might not represent the general population. Interestingly, the HIV positive rate of the age group <15 years was higher than those of the other age groups from 2006 to 2014. The reason for the higher positive rate in the age group might be a high rate of mother-to-child transmission. It was reported that most HIV-infected children under five years old had HIV-infected mothers in 1997 [46] and that one-third of new HIV infections was estimated to be caused by mother-to-child transmission [27]. The study on HIV-positive children <15 years old in Cambodia during 2003–2007 reported that the mortality after starting ART was low but the number of deaths occurring before starting ART was high because of delays in ART initiation. The rate of lost to follow-up in HIV-positive children, especially pre-ART children, was also intolerably high [47]. Finally, the improvement of the coverage of HIV testing and ART among pregnant women contributed to the decrease in the HIV positive rate in the age group <15 years old [27].

This study has some limitations. We did not analyze HIV knowledge or AIDS stigma among the sample, although lack of HIV knowledge and fear of stigma and discrimination are reported to be associated with low compliance with HIV testing. The secondary analysis of the CDHS 2010 data showed that a lower level of AIDS stigma, but not HIV knowledge, was associated with knowledge of HIV status among the general population, including men and women [28]. However, the score or level of knowledge and attitude of participants can be influenced by evaluation methods or questions. On the other hand, data on sociodemographic and behavioral factors that were used in the multivariable logistic regression in this study are subjective. Second, the prevalence of HIV testing using the CDHS data was calculated using the lifetime HIV testing rate, yet the rate in the more recent survey might be higher than that of the previous survey because of accumulation over time. However, the prevalence in the age group of 15–19 can be considered to have actually increased from 2.5% in 2005 to 7.5% in 2014, because men aged 15–19 years were not included in the previous CDHS. The third limitation is the possibility of uncertain findings caused by memory bias and embarrassment to the interviewers. Participants might not have provided honest answers to the interviewers about sexual behavior or STI-related symptoms.

## Conclusions

This study showed that the lifetime prevalence of HIV testing increased from 2006 to 2014 among the general male population in Cambodia, although the number of men who had HIV testing at the VCCT sites increased from 2006 to 2010 and decreased from 2012 to 2015. Factors associated with ever having an HIV test among the general male population in CDHS 2005–2014 were CDHS year, age group, residence, educational level, wealth index, occupation, marital status, number of lifetime sexual partners, and condom use during the last sexual intercourse. These results suggest that HIV prevention programs at primary schools throughout the country could contribute to the overall prevalence of HIV testing in Cambodia.
